# Reimagining malaria: five reasons to strengthen community engagement in the lead up to malaria elimination

**DOI:** 10.1186/s12936-015-0931-9

**Published:** 2015-10-16

**Authors:** Maxine Whittaker, Catherine Smith

**Affiliations:** University of Queensland School of Public Health, Herston, QLD Australia; Asia Research Institute, National University of Singapore, Singapore, Singapore

**Keywords:** Community engagement, Community participation, Collaboration, Malaria elimination

## Abstract

Although community engagement has been recognized as an important element of public health since the Alma Ata declaration, in practice community engagement has played a marginal role within malaria control programmes. As more countries move toward elimination, malaria elimination programmes will need to reimagine malaria in a number of ways. An important element of this will be to re-conceptualize and better strategize community engagement, which will become increasingly important for programme success as countries near elimination. This commentary intends to begin a conversation on re-imagining community engagement in an elimination setting, by outlining five ways that community engagement should be strengthened and re-strategized in the lead up to malaria elimination.

## Background

As the contributors to this thematic series point out, there are a number of ways in which malaria should be reimagined in the lead up to elimination. Although community engagement has long been recognized as a factor contributing to the successful control and elimination of many infectious diseases, including malaria, in reality community engagement has often played a marginal role within malaria control and elimination programmes in the last 15 years. This commentary points out five ways that community engagement should be re-imagined, strengthened and re-strategized in the lead up to malaria elimination. In the control era, a large volume of social science literature discussed the benefits of community participation to malaria control, and some of these benefits still apply in an elimination setting. In the control era, community participation was seen as a means to: improve community uptake of prevention, diagnosis and treatment [1, 2]; understand how local knowledge, belief and practice might influence the effectiveness of interventions [3–6]; support health promotion [7, 8]; add local knowledge to programmes [1, 2]; strengthen a primary health care approach to malaria control [2]; and increase equity within elimination and support the development outcomes of public health programmes [1, 2, 9].

However while the concept of community participation was widely accepted during the control era and was noted as a key element of Primary Health Care in the Alma Ata declaration of 1978, some have noted that malaria programmes did not fully utilize the benefits of community engagement [10, 11] especially as the pendulum swung in the MDGs period towards top-down approaches to malaria in many settings. For example, it has been argued that most ‘community engagement’ activities within malaria control in Africa were not aimed at community empowerment but rather more closely resembled top-down interventions that were motivated by a desire to bring about patient compliance [11]. A systematic review of community participation in infectious disease control programmes confirms that malaria control only partially implemented the principles of community engagement [10]. While agreeing with critics that community engagement should not be seen as a panacea to compensate for limitations in health systems or technology [12], systematic reviews of successful infectious disease control and eradication programmes show that community engagement has often been a critical factor enabling successful infectious disease control and elimination programmes [10, 13].

This commentary suggests that while community engagement is rarely recognized as an important element of malaria elimination, that in practice a large number of activities are already implemented at the community level that depend on support from communities for success. Community engagement involves a wide range of activities along a continuum, ranging from passive community acceptance at one end, to community ownership of elimination at the other [14]. The authors argue that malaria elimination will be enriched if these existing activities are recognized as a form of community engagement and incorporated into elimination planning in a more strategized manner. The authors argue that while many features of community engagement from the control era also apply to malaria elimination, that elimination also presents new challenges that make active community participation even more important for programme success. This commentary is not an exhaustive discussion of these issues, but rather intends to begin this conversation by outlining five key reasons why elimination efforts will benefit from re-imagining, strengthening and re-strategizing community engagement.

## Malaria may become less of a priority of communities in an elimination setting

One of the most compelling reasons for strengthening community engagement within elimination is that programmes will increasingly find themselves working in communities with changed perceptions about malaria and their personal risks. In such a context, it is reasonable to assume that community uptake of prevention and treatment measures may. Changes in perceptions of risks from childhood immunizable infectious diseases have adversely affected immunization uptake in many settings. It may occur that as communities become aware that few people now become sick from malaria [15] adherence to prevention activities or early recognition of malaria symptoms my be negatively affected. Atkinson and colleagues point out that the most common factor undermining community confidence in disease control programmes is a lack of perceived risk of disease. Since perceived risk lessens as diseases begin to disappear, it follows that programmes must become more proactive in both understanding the reasons behind any changes in perceptions, and formulating appropriate health promotion responses to support ongoing prevention, surveillance and response and treatment activities [10].

Malaria elimination programmes should become much more proactive in generating community support for the goal of elimination, since even when malaria is an immediate risk for individuals socio-economic factors often lead people to deprioritize malaria prevention and treatment. This is common, for example, when poverty is a barrier to the purchase of treatment measures [16], or where people choose to seek a livelihood in occupations that may increase their exposure to malaria but which are necessary to support their families [16]. As malaria continues to decline and become less immediately relevant to the lives of ordinary people, there is a potential that such factors will have more impact on programme effectiveness. In more extreme situations, communities have in the past even actively boycotted programmes that were targeted at a disease that was not a local priority. This is more likely to occur in situations where public health authorities are mistrusted or if there are concerns about the safety of interventions [17, 18].

While the ultimate goal of malaria elimination is to make malaria irrelevant to the lives of ordinary people by eliminating the disease, this perceived irrelevance should also be recognized as a challenge that programmes should be prepared to face in the near future. The point should not be to claim that malaria is the most pressing health concern to communities that in reality face many complex health issues. Rather a more beneficial strategy would be to effectively engage with communities to emphasize the positive effects that malaria elimination has already had on their communities, to explain the reasons behind ongoing malaria interventions in contexts where malaria is low, and to show communities what they can do to help stop malaria from returning and to help eliminate the disease completely.

## Many elimination activities require active community participation, rather than passive community compliance

**Fig. 1:**
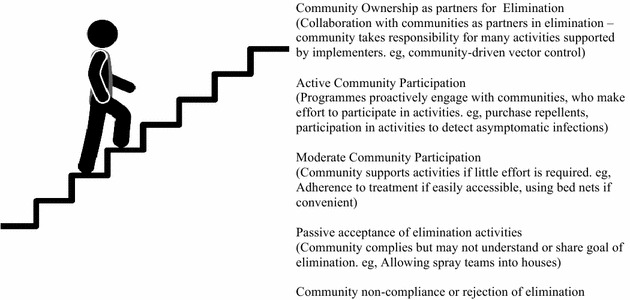
Degrees of community participation on the path to elimination

Community participation can be understood along a sliding scale, ranging from passive community compliance at one end, to highly active forms of community participation, to the eventual goal of community ownership of elimination in partnership with implementers (Fig. 1) [11]. Some have suggested that malaria programmes have often understood the goal of community participation as something resembling patient compliance, so that historically it was often seen to be sufficient to ensure passive community conformity to programme priorities [11, 14]. Examples of common malaria interventions that involve passive community acceptance might include a family allowing a spray team access to their house, or a person receiving a free bed net that is brought to them easily through a well-functioning distribution channel.

However passive acceptance is not sufficient for the success of many established malaria interventions. Examples of activities requiring a moderate level of community participation might include a patient adhering to a drug treatment protocol, a family purchasing a mosquito net or repellents, wearing protective clothing or sleeping under a mosquito net even when it may be inconvenient to do so. Where poverty or competing priorities are a factor then more proactive effort is needed on the part of elimination programmes to encourage communities to take on the malaria interventions, to ensure that they have an impact. In addition, it has long been accepted that community engagement is important where cultural understandings of febrile diseases lead to treatment-seeking practices that may be detrimental to programme success [1–10].

As more countries near elimination it will be increasingly important for communities to participate actively, rather than passively, in elimination activities. Elimination programmes should proactively engage with communities, and should begin steadily intensifying community engagement and working towards community ownership of elimination in partnership with implementers. Active community engagement is particularly important in cases where programmes wish to detect and treat asymptomatic infections, where programmes are working with groups that have historically been seen as ‘hard-to-reach’ by programmes, and in communities where the perceived risk of malaria diminishes [15, 19, 20]. Activities that require active community participation for success include community-driven vector control, or participation in mass drug administration [20]. As malaria continues to reduce in many countries, programmes should begin to strengthen their community engagement activities in preparation for these emerging challenges.

## Community engagement can support programme implementation at the subnational and community level

Although community engagement has not historically formed a strong component of malaria control and elimination strategy, in practice many malaria control programmes have benefitted from community-level activities that work as crucial mechanisms enabling programme implementation. Kuhlmann and Ianotti recently noted that the strong emphasis given to the trans-border and global nature of public health issues detracts attention from the fact that much programme implementation still occurs at the national, subnational or community level [21]. Programmes will benefit from recognizing that many malaria elimination activities already have some component of community engagement that can be strengthened to support programme implementation at the sub-national and community level.

For example, community-driven vector control relies on high levels of active community participation, and is considered to have played a role in programme success in many countries. In the Philippines for example, effective relationship building between malaria officers, village (*barangay*) leaders and community health workers helped to mobilize the broader community and strengthened community-driven vector control [22]. This was particularly valuable in an elimination setting where vector control needed to be stratified to the micro level. This community engagement built on earlier successes with community health workers, who had already established effective relationships with local leaders and the community. By linking vector control with an already established mechanism for community engagement (the village health worker programme) malaria officers were able to build valuable relationships with communities and local government and accelerate programme implementation [22–25].

Other well established malaria control and elimination activities that depend strongly upon effective community engagement include community health worker programmes [26, 27], health promotion and behaviour change communication activities [7, 8], and surveillance and response activities [28]. While their primary goal is not community engagement in itself, these activities are often the point of interaction between programmes and communities so that they strongly shape community perceptions of malaria elimination programmes. This also makes these programmes an ideal point of entry as programmes develop more strategized community engagement, as well as supporting important community-level elimination activities. Strategizing community engagement focuses on clearly identifying, for various parts of the elimination programme and in the particular socio-economic and cultural contexts, the outcomes desired from the engagement and local appropriate ways to achieve those outcomes. For example, for “eyes and ears” surveillance community members develop appropriate ways to encourage people entering their community to protect themselves from malaria, seeks early diagnosis and treatment if they have a fever or continue their treatment activities [29]. If the prevention of malaria epidemics depends on larval source management in that community context, engaging communities to find solutions to monitoring larval numbers and applying appropriate management responses would be appropriate. By re-imagining community engagement and recognizing that it already occurs within many ongoing programme activities, countries will better able to developed strategized community engagement that helps to ensure community engagement in the goal of elimination.

## Community engagement can empower communities and make public health more equitable

Community engagement has traditionally been seen as a way to empower communities and bring about more equitable and sustainable development [1, 2, 10, 13, 14]. Much of the early writing on malaria and community participation followed the Alma Ata Declaration and occurred at a time when participatory development was a growing component of the aid sector. Advocates of community participation in malaria control often saw the empowerment of local communities both as an ethical issue that enabled greater social equity through public health, and as a pragmatic issue since it was seen that malaria control interventions would be more successful if they were in line with community priorities [10, 13, 14].

As in the control era, community engagement in elimination will help to bring about greater equity within elimination activities. This may become increasingly important as elimination activities become increasingly targeted toward people who are already marginalized in some way [30–32]. Working closely with communities will help minimize potential negative effects of interventions, e.g. stigmatization of particular groups or locations, culturally and locally inappropriate packaging of health and development activities including malaria activities. In an elimination setting, the goal should shift from ensuring participation to working toward community ownership of elimination, in which communities see themselves as sharing a stake in elimination and work with authorities to increase accessibility to a broader range of health activities which may have been unavailable before. Atkinson et al. [10] discussed the concept of harmonizing efforts to build “competent communities”, in this case for malaria elimination, can support broader accessibility to other communicable disease control services and support efforts to optimizing health system effectiveness, which this paper argues, supports the equity agenda.

## Community engagement will help to prepare for the prevention of reintroduction phase

Malaria elimination has progressed rapidly over the past decade and more countries are nearing elimination and working toward the prevention of reintroduction [33]. In the prevention of reintroduction phase countries need to continue activities to respond to outbreaks and prevent resurgences. The activities that countries will need to continue after they achieve elimination depend upon a number of factors including the intrinsic receptivity of the area, the risk of imported malaria, the strength of health systems to treat isolated infections, and the capacity for case surveillance and response to prevent an outbreak becoming an epidemic [34].

Strong community engagement in the prevention of reintroduction phase can support countries to carry out ongoing activities in a context where the risk of disease to individuals is greatly reduced, but where the costs of a resurgence is very high, due to decreased immunity. It is generally accepted that advocacy and leadership for elimination is vital to ensure sustained political commitment and funding for malaria in an elimination setting [35]. Likewise, effective community engagement is necessary to explain the reasoning behind continued activities and to ensure community participation in activities at the very minimum.

As stated above however, elimination will be greatly strengthened if programmes re-imagine community engagement not only as a means for ensuring passive community acceptance, but as a method for building active community engagement with the goal of eventually achieving community ownership of elimination. Rather than waiting until the prevention of reintroduction phase, strong community engagement should begin in the pre-elimination and elimination phases, as this will help programmes to prepare for prevention of reintroduction activities. Ongoing community engagement will progressively build community involvement in elimination activities, improve an understanding of malaria and the reasoning behind elimination activities, and build awareness of the possibility of outbreaks and resurgences. If programmes achieve community ownership of elimination before they reach the prevention of reintroduction phase, they will be much more likely to transfer responsibility and costing for some ongoing activities to individuals and communities.

## Conclusion

This commentary has argued that malaria elimination programmes will greatly benefit from re-imagining both the process and the purpose of community engagement activities in the lead up to elimination. Community engagement is not a standalone activity, but an element of many existing activities that are implemented at the community level. As malaria disappears from communities and has a diminishing or less direct impact on people’s lives, programmes should become more proactive in generating community support for elimination activities, and in building a broader awareness of ongoing malaria risk despite reductions in disease, as it is observable to ordinary people in the course of their lives. While malaria elimination programmes have often in the past been satisfied with passive community conformity to programme activities, many elimination activities require much higher levels of active community participation. Elements that increase community engagement include: increased knowledge at individual and community level of the disease, its causality, prevention & treatment; working with communities to develop acceptable effective intervention packages; understanding and addressing the community and household incentives and disincentives for participation e.g. opportunity costs; gendered and cultural sensitive approaches to participation and engagement; working with the levels of social cohesion; commitment of authorities to genuine participation and decentralization of decision making; appropriate levels of support and resources for participation and locally embedded civil society agencies [10].

By re-imagining, strategizing and strengthening community engagement in the pre-elimination stage, countries will be better prepared to ensure community support for activities as they near elimination and begin prevention of reintroduction activities.
